# Retrospective epidemiological analysis of pulmonary tuberculosis in the older adult and characterization of rifampicin resistance-associated *rpoB* mutations in Nantong City, China (2014–2023)

**DOI:** 10.3389/fpubh.2025.1577211

**Published:** 2025-05-26

**Authors:** Huiming Zhu, Shouwei Wang, Xiaofang Zhao, Peipei Wu, Ji Chen, Feng Li

**Affiliations:** ^1^Department of Clinical Laboratory, Affiliated Nantong Hospital of Shanghai University (The Sixth People's Hospital of Nantong), Nantong, China; ^2^Department of Public Health, Affiliated Nantong Hospital of Shanghai University (The Sixth People's Hospital of Nantong), Nantong, China; ^3^Qinzao Health Service Center of Nantong, Nantong, China; ^4^Department of Chronic Infectious Diseases, Nantong Center for Disease Control and Prevention, Nantong, China

**Keywords:** *Mycobacterium tuberculosis*, older adult pulmonary tuberculosis, rifampicin resistance, *rpoB* gene mutation, retrospective analysis

## Abstract

**Objective:**

To analyze the prevalence trend of older adult pulmonary tuberculosis (ETB) and the distribution and outcome of rifampicin-resistant *rpoB* gene mutation in ETB patients in Nantong.

**Methods:**

The pulmonary tuberculosis patients’ data in Nantong from 2014 to 2023 were from Tuberculosis Information Management Syste and ETB and rifampicin-resistant *rpoB* mutation patients were retrospectively analyzed.

**Results:**

From 2014 to 2023, the overall standardized incidence of ETB in Nantong showed a trend of rapid decline and tended to a stable trend stabilized. A total of 140 older adult patients with rifampin resistance, aged 60–69 years, 87 cases (62.1%). single-gene mutation Probe E mutations were the most frequent, observed in 39 cases (60.0%). Specifically, 52 cases (80.00%) were resistant to rifampicin, and Probe E of 31 cases (59.62%) showed the most mutations. The outcome of ETB patients with rifampicin resistance were significantly correlated with treatment classification, rifampicin resistance, Xpert MTB first test, and 0-month sequential sputum positivity.

**Conclusion:**

The number of ETB in Nantong from 2014 to 2023 showed a rapid decline and stabilized. The *rpoB* mutations in the ETB rifampicin-resistant patients were mainly single-gene mutations. The authorities should formulate effective regional prevention and control measures based on the characteristics of the ETB rifampicin-resistant patients.

## Introduction

Tuberculosis (TB), which is one of the top ten causes of death in the world, has caused a major public health problem, with its morbidity and mortality showing a significant upward trend (1). TB is a chronic infectious disease (class B infectious disease) caused by *Mycobacterium tuberculosis* (MTB) complex group infection of the body. MTB can infect various organs of the body, the most common of which is to invade the lung, which is called pulmonary tuberculosis (PTB) (2–4). The important source of tuberculosis infection is bacterial-positive patients (bacterial-discharging patients), and the body may not become sick after being infected by the tuberculosis bacteria. However, the incidence will be greatly increased when the immunity is reduced.

Drug-resistant tuberculosis poses a serious threat to human health and has become a difficult and key issue in TB control (5). According to the World Health Organization’s Global Tuberculosis Report 2020, there were an estimated 10 million new cases of tuberculosis worldwide in 2019, and about 3.3% (465,000 cases) of newly treated patients and 18% of retreated patients were resistant to rifampicin, of which about 78% (363,000 cases) were multidrug-resistant tuberculosis (MDR-TB) patients (6).

According to relevant studies at home and abroad, the mutation of the rifampin resistance determining region (RRDR) in the *rpoB* gene of MTB is closely related to 95% of rifampicin-resistant cases (7–9). Rifampicin cannot be firmly bound to the *β* subunit of DNA-dependent RNA polymerase, and cannot inhibit the synthesis of *mycobacterium tuberculosis* RNA, so it loses its bactericidal effect (10, 11).

Studies have shown that older adult patients with tuberculosis are characterized by low immune function, irregular medication and delayed medical treatment, etc., and have become a population with a high incidence of tuberculosis (12, 13), which has brought severe tests to the prevention and control of tuberculosis (14). At present, there is a lack of research on the distribution and outcome of *rpoB* mutations in older adult rifampicin-resistant patients in Nantong City. In this study, we aimed to provide a basis for the prevention and treatment of older adult pulmonary tuberculosis (ETB) and rifampicin-resistant patients in Nantong city by analyzing the prevalence trend of tuberculosis in the older adult aged 60 years and above, and the distribution characteristics and outcome of rifampicin-resistant *rpoB* gene mutations.

## Materials and methods

### Data source

The research content of older adult patients with rifampicin resistance in this study has been approved by the Medical Ethics Committee of the Sixth People’s Hospital of Nantong (approval No.: NTLYLL2024019). Written informed consent for the use of samples was obtained from all participants. The data were from Tuberculosis Information Management System, the subsystem of the China Information System for Disease Control and Prevention, which collects the registration of tuberculosis patients in Nantong from 2014 to 2023, focusing on screening the case information of ETB patients aged 60 and above, and the older adult population data come from the official website of the Nantong Municipal Bureau of Statistics. From 2019 to 2023, the distribution of rifampicin-resistant *rpoB* gene mutations in patients with rifampicin resistance was sourced from Nantong Tuberculosis Designated Treatment Hospital.

### Related definitions

The patients with tuberculosis refer to the suspected tuberculosis patients reported and registered by each county and district. The symptoms, imaging characteristics and epidemiological history of the patients are judged by the experienced specialists in the designated tuberculosis hospitals. Only when the laboratory results are highly consistent with the clinical diagnosis, the patients who are finally confirmed to be effective and entered into the system.

Tuberculosis pathogen positive refers to tuberculosis patients found by tuberculosis smear, culture and molecular biology methods. The source of patients refers to the way of tracing the patient ‘s treatment through direct treatment, referral, tracking, physical examination and promotion. The definition of tuberculosis resistance and rifampicin resistance refers to the “Guidelines for the Management of Drug-resistant Tuberculosis Planning “issued by the World Health Organization (WHO) (15).

In this study, probe A-E corresponded to a specific codon region in the rpoB gene, in which probe E targeted the 531st codon (ATC) in the 81 bp core region of the rpoB gene, which is a key mutation site related to rifampicin resistance. By specifically binding to this region, it is used to detect the base variation of rpoB gene at this site. Joinpoint regression is a statistical method commonly used in time series data analysis. It is mainly used to identify the time point (i.e., turning point) where the data trend changes, and to quantitatively analyze the trend of different stages. In terms of model selection, we use Akaike Information Criterion (AIC) and Bayesian Information Criterion (BIC) to determine the optimal number and location of Joinpoints to ensure the goodness of fit of the model. By calculating indicators such as Annual Percentage Change (APC) and Average Annual Percentage Change (AAPC), the trend changes at different stages are quantitatively analyzed, which provides objective indicators for trend comparison and evaluation.

### Instruments and reagents

Real-time fluorescence PCR detector (Xpert MTB/RIF, Cepheid, United States) and supporting reagents, mycobacterial antimicrobial susceptibility Roche culture tubes are provided by Zhuhai Besso Biotechnology Co., Ltd. Results determination and interpretation were as follows:

Xpert MTB check: The sputum or lavage samples were transferred to the sample management tube, and 4%NaOH of 1–2 times the volume was added. The samples were shaken for 1 min, then fully liquefied and left for 15 min at room temperature. 2 mL of liquid was absorbed and added to the reaction box for ultrasound, lysis, fluorescence amplification, polymerase chain and DNA reflection. The device automatically interprets the results of the TB test, whether it is resistant to rifampicin, and the mutation of the rifampicin-resistant rpoB gene. In this study, the Xpert MTB / RIF detection system was used to detect the rpoB gene mutation, and the interpretation criteria of the “Guidelines for the Management of Drug-resistant Tuberculosis Planning “issued by the World Health Organization (WHO) were strictly followed. The specific decision rule is: when the detection cycle threshold (Ct value) ≤ 38 and the rpoB gene detection result is positive, combined with the fluorescence signal intensity of the 531 codon region where the probe E is located, if the signal is abnormal, it indicates that there is a mutation at this site; at the same time, all positive results were reviewed twice, and the mutation sites were verified by pyrosequencing technology to ensure the accuracy of the test results.

Roche ratio method: The fresh colonies were selected by inoculation, ground in the fungus mill bottle, and compared with the Machelloway turbidimetric tube to produce 1 mg /mL bacterial suspension, then diluted 100 times to obtain 10–2 mg /mL bacterial solution, and then diluted to 10–4 mg /mL to obtain two concentrations of working bacterial solution. The two bacterial liquids were inoculated on the incline of the drug-containing medium, and the drug-sensitive results were observed after incubation at 37°C for 1 month: if the drug resistance detection result exceeded 1%, it was drug-resistant; if the number of colonies in the high-dilution blank control medium was less than 20, the test should be repeated.

### Observation indicators

The results of the seventh census of Nantong City in 2020 were used as the standard population, the standardized incidence of tuberculosis in the older adult aged 60 years and above was analyzed, and the trend of gender composition and age segment of the older adult in each year was analyzed and observed. Meanwhile, rifampicin resistance and *rpoB* gene mutation of rifampicin resistance in the older adult were analyzed.

### Statistical analysis

Excel 2017 and SPSS27.0 software were used for data processing and data analysis. Joinpiont 5.0.1 software was used to analyze the trend of registration rate of older adult patients with time, and the annual percentage change (APC) and average annual percentage change (AAPC) were calculated. The normally distributed continuous data were used ^−^x ± s, and the t-test was used between the two groups, and *p < 0.05* was considered statistically significant.

## Results

### Basic situation of incidence registration and standardized incidence of ETB patients

From 2014 to 2023, a total of 11,498 patients aged 60 and above with PTB were registered in Nantong City, and the number of reported cases decreased from 1,607 in 2014 to 915 cases in 2023, the highest in 2015 (1,652 cases) and the lowest in 2022 (831 cases). The standardized incidence showed a trend of decreasing first and then stabilizing, with the highest standardized incidence rate in 2015(71.25/100,000) and the lowest in 2022 (35.84/100,000) (Table 1).

**Table 1:** Register form of the incidence of ETB patients in Nantong City (2014–2023).

Year	Total		Gender					Age				
			Male		Female			60–69	70–79		≥80	
	NC *n*	ASIR (/lakh)	NC *n*	ASIR (/lakh)	NC *n*	ASIR (/lakh)	NC *n*	ASIR (/lakh)	NC *n*	ASIR (/lakh)	NC *n*	ASIR (/lakh)
2014	1,607	69.31	1,172	104.39	435	36.37	748	65.20	592	76.73	267	66.75
2015	1,652	71.25	1,222	108.84	430	35.95	756	65.90	614	79.59	282	70.50
2016	1,436	61.93	1,044	92.99	392	32.78	628	54.74	572	74.14	236	59.00
2017	1,243	53.61	927	82.57	316	26.42	582	50.73	449	58.20	212	53.00
2018	1,094	47.18	840	74.82	254	21.24	510	44.46	387	50.16	197	49.25
2019	985	42.48	734	65.38	251	20.99	466	40.62	337	43.68	183	45.75
2020	900	38.81	689	61.37	211	17.64	402	35.04	337	43.68	161	40.25
2021	834	35.97	666	59.32	168	14.05	370	32.25	297	38.50	167	41.75
2022	831	35.84	651	57.99	180	15.05	314	27.37	325	42.13	192	48.00
2023	915	39.46	716	63.77	199	16.64	394	34.34	345	44.72	176	44.00

NC, Number of cases; ASIR, Standardized rate.

### Trend of reported standardized incidence in ETB patients

The results of Joinpoint regression model analysis showed that the average annual standardized incidence of ETB in Nantong decreased by 6.91% (95%CI, −9.85 ~ −3.87; *t* = −4.37, *p* < 0.001), and presented different trends in the two periods. From 2014 to 2021, this segmentation showed it decreased by 10.15%, a significant decline, while from 2021 to 2023, it showed an increase by 5.39%, tending to be stable.

The average annual decline in male patients was 6.09% (95%CI: −9.11 ~ −2.97; *t* = −3.77, *p* < 0.001), Among them, the sub-segment showed a significant decline of 9.16%from 2014 to 2021, and while increased by5.50% from 2021 to 2023, tending to be stable. The average annual decrease in female patients was 9.44% (95%CI: −13.56 ~ −5.13; *t* = −4.18, *p* < 0.001), the segmentation showed also a significant decline by 15.59% from 2014 to 2021, and while increased by 5.04% from 2021 to 2023, tending to be stable.

**Figure 1:**
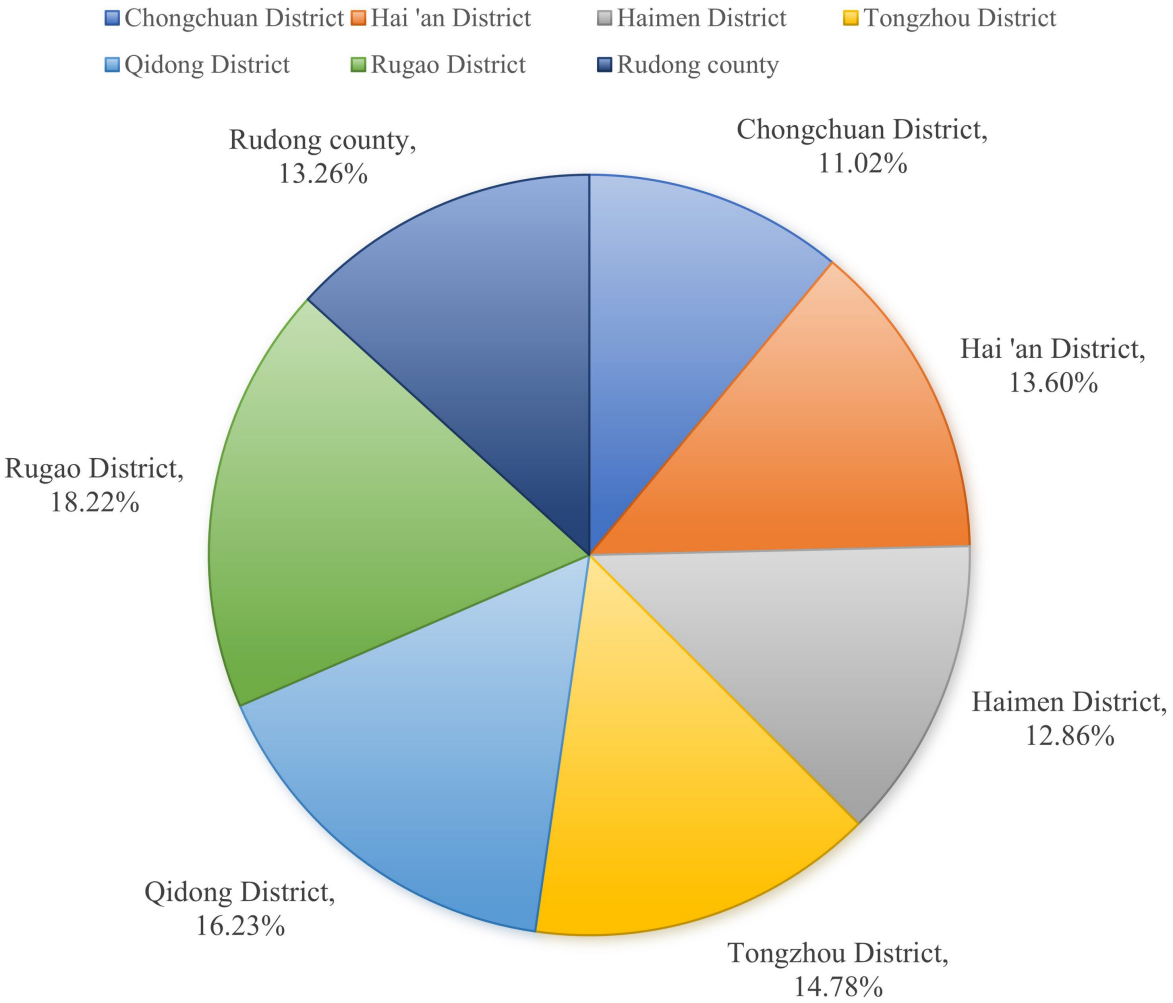
Trend chart of reported incidence rate of ETB patients in Nantong City.

Patients aged 60–69 years decreased by an average of 8.31%per year (95%CI: −10.85 ~ −5.69; *t* = −6.05, *p* < 0.001). Among them, the sub-segment showed a significant decline of 10.82% from 2014 to 2021, with a significant decline, and increased by 1.06% from 2021 to 2023, which tends to be stable. Patients aged 70–79 years decreased by an average of 6.58% per year (95%CI: −11.65 ~ −1.21; *t* = −2.39, *p* = 0.017). the segmentation showed that it decreased by 10.69% from 2014 to 2021, with a significant decline, with a significant decline, and increased by 9.35% from 2021 to 2023, tending to be stable. Patients aged 80 years and older decreased by an average of 4.69% per year (95%CI: −7.98 ~ −1.28; *t* = −2.68, *p* = 0.007), the segmentation shows that it decreased by 8.64%from 2014 to 2021, with a significant decline, and increased by 3.74% from 2021 to 2023, tending to be stable (Table 2; Figure 1).

**Table 2:** A Joinpoint retrospective analysis of the trend of the reported incidence rate of ETB patients in Nantong City (2014–2023).

Characteristic	Year	APC (95%CI)	*T* value	*p* value	AAPC (95%CI)	*T* value	*p* value
Total	2014–2021	−10.15* (−12.19, − 8.06)	8.06	<0.001	−6.91* (−9.85, −3.87)	−4.37	<0.001
	2021–2023	5.39 (−11.24, 25.14)	0.79	0.47			
Gender							
Male	2014–2021	−9.16* (−11.26, −7.02)	−10.58	<0.001	−6.09* (−9.11, −2.97)	−3.77	<0.001
	2021–2023	5.50 (−11.41, 25.65)	0.79	0.47			
Female	2014–2021	−13.20* (−16.04, −10.27)	−10.95	<0.001	−9.44* (−13.56, −5.13)	−4.18	<0.001
	2021–2023	5.04 (−18.09, 34.70)	0.51	0.63			
Age							
60–69	2014–2021	−10.82* (−12.59, −9.01)	−14.66	<0.001	−8.31* (−10.85, −5.69)	−6.05	<0.001
	2021–2023	1.06 (−13.04, 17.43)	0.18	0.86			
70–79	2014–2021	−10.69* (−14.18, −7.05)	−7.29	<0.001	−6.58* (−11.65, −1.21)	−2.39	0.017
	2021–2023	9.35 (−18.85, −47.36)	47.36	0.77			
≥80	2014–2020	−8.64* (−12.11, −5.04)	−6.01	0.002	−4.69* (−7.98, −1.28)	−2.68	0.007
	2020–2023	3.74 (−7.48, 16.32)	0.82	0.45			

APC, annual percentage change; AAPC, average annual percentage change.

### Distribution of ETB patients

**Figure 2:**
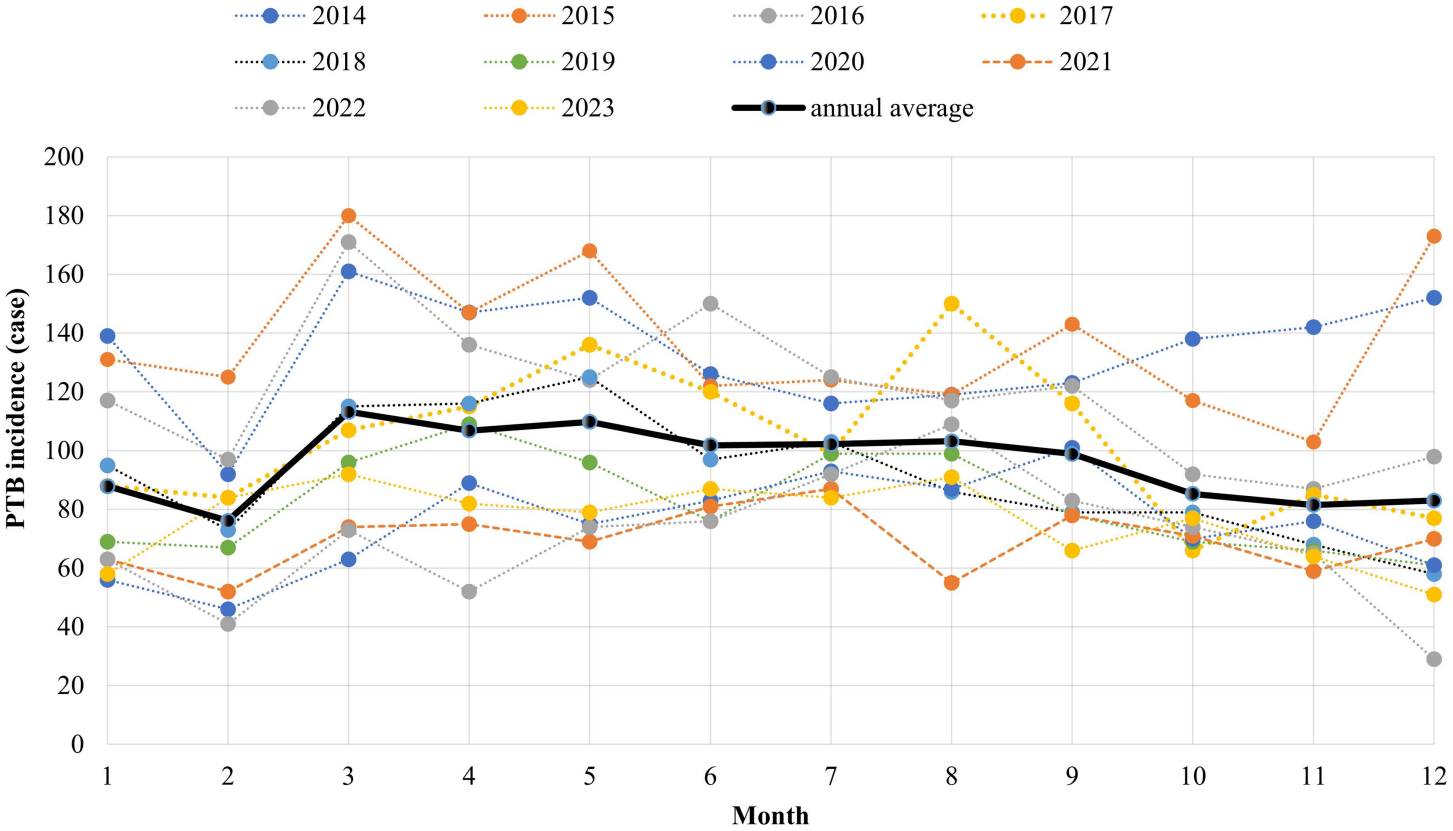
Monthly distribution of ETB patients in Nantong City (2014–2023).

**Figure 3:**
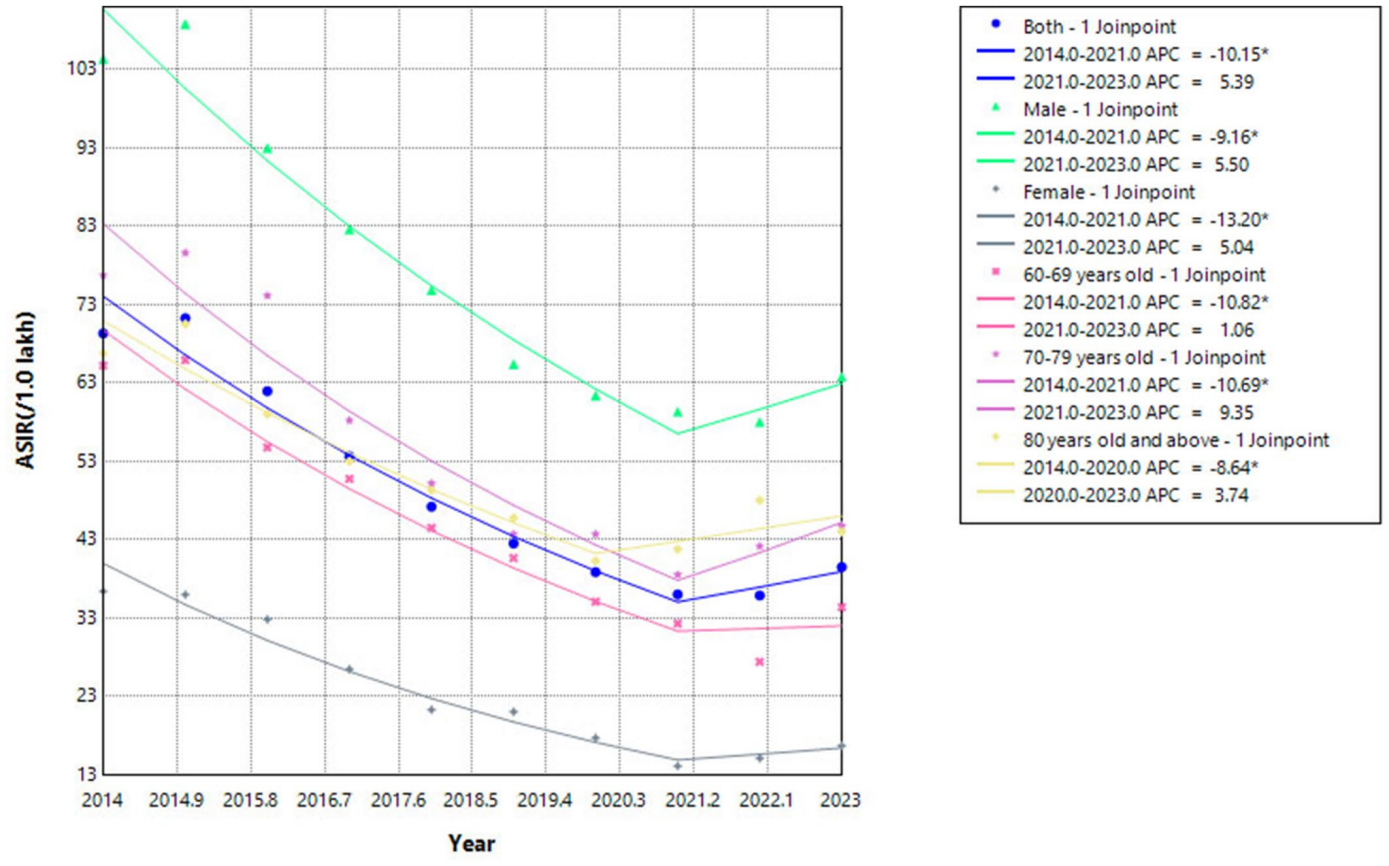
Proportion of ETB patients in each county and district of Nantong City (2014–2023).

From 2014 to 2023, the ETB patients in Nantong were mainly gathered in Rugao, Qidong and Tongzhou county, accounting for 18.22, 16.23 and 14.78%, respectively. The peak incidence of patients was mainly concentrated from March to September every year, and the incidence trough appeared in February (Figures 2, 3).

### Comparison of the characteristics of ETB patients resistant to rifampicin and non-drug resistance

From 2014 to 2023, there were a total of 4,304 ETB patients, including 140 rifampicin-resistant patients and 4,164 non-resistant patients, who were divided into drug-resistant group and non-drug-resistant group, all of whom were aged 60–69 years, male, farmers, local household registration and referred patients, The age of 60–69 years old (t/x2 = 22.471, *p* < 0.05), population classification (t/x2 = 16.090, *p* < 0.05), floating population (t/x2 = 4.496, *p* < 0.05), referral (t/x2 = 19.418, *p* < 0.05), retreatment patients (t/x2 = 103.595, *p* < 0.05) in the rifampicin-resistant group were significantly higher than those in the non-resistant group, and the differences were statistically significant. There was no significant difference in gender (t/x2 = 3.647, *p* > 0.05), diabetes mellitus (t/x2 = 4.104, p > 0.05) and the use of fixed-dose procedure (FDC) (t/x2 = 0.425, *p* > 0.05) between the two groups (Table 3).

**Table 3:** Characteristics of rifampicin-resistant and non-resistant ETB patients in Nantong City (2014–2023).

Classification	Drug-resistance *n* = 140	Non-drug resistance *n* = 4,164	t/x^2^	*p* value
Age			22.471	<0.001
60–69	87 (62.10)	1821 (43.70)		
70–79	44 (31.40)	1,585 (38.10)		
≥80	9 (6.40)	758 (18.20)		
Gender			3.647	0.060
Male	105 (75.00)	3,399 (81.60)		
emale	35 (25.00)	765 (18.40)		
Classification			16.090	0.003
Worker	4 (2.86)	30 (0.72)		
Unemployed	4 (2.86)	41 (0.98)		
Retirement	14 (10.00)	567 (13.62)		
Farmer	116 (82.86)	3,506 (84.20)		
Other	2 (1.42)	20 (0.48)		
Diabetes			4.104	0.128
Yes	3 (2.30)	85 (2.00)		
No	94 (73.40)	3,354 (80.50)		
Uncertainty	31 (4.10)	725 (17.40)		
Census register			4.496	0.034
Local population	130 (92.90)	4,024 (96.60)		
Floating population	10 (7.10)	140 (3.40)		
Patient source			19.418	<0.001
Direct visit	29 (20.70)	933 (22.40)		
Transfer treatment	62 (44.30)	1892 (45.40)		
Trace	44 (31.40)	1,316 (31.60)		
Referrals	2 (1.40)	8 (0.20)		
Physical examination	3 (2.10)	15 (0.40)		
Treatment classification			103.595	<0.001
Initial treatment	60 (42.90)	3,422 (82.20)		
Retreatment case	80 (57.10)	742 (17.80)		
Using FDC			0.636	0.425
Yes	58 (41.40)	1867 (44.80)		
No	82 (58.60)	2,297 (55.20)		

FDC, fixed-dose procedure.

### Distribution of *rpoB* gene mutations of rifampicin resistance in ETB patients

Among the 65 older adult rifampicin-resistant patients, 64 (98.46%) had a single gene mutation and 1 (1.54%) had a double gene mutation. The highest single gene mutation was Probe E, with 39 cases (60.00%), followed by Probe D, with 14 cases (13.86%). The double gene mutation was Probe A + B, 1 case (1.54%); In the older adult group and non-older adult group (t/x2 = 4.320, *p* < 0.05), initial treatment and retreatment group (t/x2 = 16.023, *p* < 0.05), Probe E was statistically significant, and the rest were not statistically significant Tables 4, 5.

**Table 4:** Distribution of rpoB gene mutations in older adult and non-older adult rifampicin in Nantong City (2019–2023).

Classification of patients	Type of probe						
	ProbeA (507–511)	ProbeB (512–518)	ProbeC (518–523)	ProbeD (523–529)	ProbeE (529–533)	ProbeA+B (507–518)	ProbeA+E (507–511) + (529–533)
Older adult (*n* = 65)	6 (9.23)	5 (7.69)	0	14 (21.54)	39 (60.00)	1 (1.54)	0
Non-older adult (*n* = 101)	5 (4.95)	2 (1.98)	2 (1.98)	14 (13.86)	76 (75.25)	1 (0.99)	1 (0.99)
*x* ^2^	0.242	1.937		3.250	4.320		
*p*	0.623^b^	0.164^b^	0.521^a^	0.071	0.038	1.000^a^	1.000^a^

^a^Fisher exact probability method was used. ^b^Continuous correction Chi-square test was used.

**Table 5:** Distribution of rpoB gene mutations of rifampicin in the older adult after initial and retreatment.

Classification of patients	Type of probe						
	ProbeA (507–511)	ProbeB (512–518)	ProbeC (518–523)	ProbeD (523–529)	ProbeE (529–533)	ProbeA+B (507–518)	ProbeA+E (507–511) + (529–533)
Initial treatment (*n* = 28)	2 (7.14)	2 (7.14)	0	8 (28.57)	12 (42.86)	0	0
Retreatment (*n* = 37)	4 (10.81)	3 (8.11)	0	6 (16.22)	27 (72.97)	1 (2.70)	0
*x* ^2^	0.005	0.000		1.440	6.023		
*p*	0.942^b^	1.000^b^	–	0.230	0.014	1.000^a^	–

^a^Fisher exact probability method was used. ^b^Continuous correction Chi-square test was used.

Of the 65 drug-resistant patients, 52 (80.00%) were resistant to rifampicin only, and Probe E was the most mutated site, accounting for 31 cases (59.62%), followed by Probe D, 11 (21.15%). There were 13 cases (20.00%) of multidrug resistance, and the most mutation was Probe E, 8 cases (61.54%), followed by Probe D, 3 cases (23.08%). There was no significant difference in mutation sites between single rifampicin resistance and polydrug resistance (*p* > 0.05) (Table 6).

**Table 6:** Analysis of mutation sites of rifampicin resistance and multidrug resistance.

Classification of patients	Type of probe						
	ProbeA (507–511)	ProbeB (512–518)	ProbeC (518–523)	ProbeD (523–529)	ProbeE (529–533)	ProbeA+B (507–518)	ProbeA+E (507–511) + (529–533)
Rifampin-resistance (*n* = 52)	5 (9.62)	4 (7.69)	0	11 (21.15)	31 (59.62)	1 (1.92)	0
Multi drug resistance (*n* = 13)	1 (7.69)	1 (7.69)	0	3 (23.08)	8 (61.54)	0	0
*x* ^2^	0.000	0.000		0.000	0.016		
*p*	1.000^b^	1.000^b^	–	1.000^b^	0.899	1.000^a^	–

^a^Fisher exact probability method was used. ^b^Continuous correction Chi-square test was used.

### Treating outcomes of rifampicin-resistant ETB patients

Of 140 ETB rifampicin-resistant patients, 25 patients were excluded, 115 patients were analyzed, 31 cases were successfully treated, 84 cases were not successfully treated, The patients were mainly about 70 years old (t/x2 = 1.080, *p* > 0.05), male (t/x2 = 0.08, *p* > 0.05), farmers (t/x2 = 4.153, *p* > 0.05), local (t/x2 = 1.707, *p* > 0.05), referral patients (t/x2 = 0.962, *p* > 0.05), and the differences were not statistically significant. Whether the patient was retreated (t/x2 = 10.749, *p* < 0.05), multi-drug resistance (t/x2 = 5.898, *p* < 0.05), Xpert MTB first test (t/x2 = 4.968, *p* < 0.05), 0 month sputum sequence results (t/x2 = 8.550, *p* < 0.05) were statistically significant for the patient ‘s outcome (Table 7).

**Table 7:** Treatment outcomes of rifampicin-resistant ETB patients in Nantong City (2014–2023).

Classification of patients	Successful treatment *n* = 31	Unsuccessful treatment *n* = 84	t/x^2^	*p* value
Age	70.39 ± 7.25	68.94 ± 6.41	1.080	0.282
Gender			0.080	0.930
Male	23 (74.20)	63 (75.00)		
Female	8 (25.80)	21 (25.00)		
Classification			4.153	0.245
Worker	0 (0)	2 (2.40)		
Unemployed	0 (0)	2 (2.40)		
Retirement	1 (3.20)	11 (13.10)		
Farmer	30 (96.80)	69 (82.10)		
Census register			1.707	0.191
Local population	28 (90.30)	81 (96.40)		
Floating population	3 (9.70)	3 (3.60)		
Patient source			0.962	0.916
Direct visit	7 (22.60)	17 (20.20)		
Transfer treatment	13 (41.90)	38 (45.20)		
Trace	10 (32.30)	25 (29.80)		
Referrals	0 (0)	2 (2.40)		
Physical examination	1 (3.2)	2 (2.40)		
Treatment classification			10.749	0.001
Initial treatment	5 (16.10)	42 (50.00)		
Retreatment case	26 (83.90)	42 (50.00)		
Rifampin alone			5.898	0.015
Yes	25 (80.60)	47 (56.00)		
No	6 (19.40)	37 (44.00)		
Xpert MTB first test			4.968	0.026
Yes	9 (29.00)	44 (52.40)		
No	22 (71.00)	40 (47.60)		
0-month sequential sputum			8.550	0.003
Positive	30 (96.80)	60 (71.40)		
Negative	1 (3.20)	24 (28.60)		

A total of 140 rifampicin-resistant ETB patients were enrolled, of which 25 patients were excluded from treatment, and 115 cases were analyzed (2014–2023).

## Discussion

In recent years, great achievements have been achieved in the prevention and control of tuberculosis in China, and it has been effectively controlled (16). However, rifampicin-resistant and multidrug-resistant tuberculosis have become major problems that harm public health due to their difficult treatment, low cure rate and great harm (17–19).

Nantong, a southeast coastal located open city in Jiangsu, China, has always taken TB prevention and control as an important object of public health prevention and control, and has implemented a series of measures in recent years to improve the discovery, diagnosis and treatment of rifampicin and MDR patients. At the end of 2018, Gene-Xpert molecular testing equipment was equipped in Nantong designated tuberculosis hospital, which greatly improved the detection capability and screening intensity of rifampicin-resistant tuberculosis in the city, and provided a strong technical guarantee for early detection and treatment of rifampicin-resistant tuberculosis (20).

Our retrospective study found that from 2014 to 2023, Nantong City has made remarkable achievements in TB prevention and control. The overall annual standardized incidence of tuberculosis patients showed a rapid decline and then tended to be stable. The ETB patients mainly had a higher reported rate of tuberculosis in males over 60 to 69 years old. The positive rate of retreatment and etiology was significantly higher than that of non-older adult people. According to the seventh census, the aging rate of Nantong City is 30.01%, ranking first in the country, and the older adult have many basic diseases, low immunity, irregular medication, and delayed medical treatment, becoming a high incidence of tuberculosis. The patients were mainly gathered in Rugao, Qidong and Tongzhou county, accounting for 18.82, 16.23 and 14.78%, respectively, which may be due to the large population base and serious aging in these three areas. Among them, the peak of the incidence of patients is mainly concentrated in March to September every year, and the incidence of low incidence cases in February accounted for 6.62% of the whole year, which may be related to the annual Lunar New Year, the change of floating population, and the routine physical examination of older adult people over 60 years old carried out from April to October every year, promoting more TB patients to be found.

In Nantong City, the ETB rifampicin-resistant patients were mainly male patients aged 60–69 years old, farmers, local household registration, and referred patients, with a ratio of 3:1, which may be related to male bad habits, high job mobility, and low income, reminding us that future prevention and control work should strengthen detection and publicity of older adult male population, especially in their 60–69 years old. Among the 65 ETB rifampicin-resistant patients from 2019 to 2023, single gene mutation was the dominant, with 64 cases (98.46%) of single gene mutations. The highest single gene mutation was Probe E, 39 cases (60.00%), followed by Probe D,14 cases (13.86%). The results of this study were similar to those of Ethiopia and Tanzania. These similarities indicated that frequent population movements and intensive contacts in a large number of low-income countries have significantly accelerated the mixed transmission of strains and led to the spread of mutant strains within the community. However, our findings were different from those in northeastern India, which showed that the most common mutations were at probes A and D, respectively. The frequency and pattern of *rpoB* mutations may vary geographically, and some mutations are more common in specific regions or *Mycobacterium tuberculosis* lineages (7, 9, 21). Probe E had statistical significance between the older adult group and the non-older adult group, and between the initial treatment and the re-treatment group, while the others had no statistical significance. There was no significant difference in mutation sites between rifampicin resistance and multidrug resistance. The ETB rifampicin-resistant patients in Nantong were mainly about 70 years old, male, farmer, local and referred patients, and the outcomes of them were not ideal, and the outcome of patients had statistical significance with initial retreatment, multi-drug resistance, Xpert MTB first test and 0-month sequential sputum smear results.

In summary, from 2014 to 2023, the ETB patients in Nantong City showed a rapid decline and then tended to be stable. They were mainly concentrated in 60–69 years old, males, farmers and patients with retreatment, and had the characteristics of poor treatment outcomes. Attention should be more paid to the patients with retreatment. The *rpoB* gene mutation was mainly a single gene mutation, with the highest is Probe E, which showed no difference with the mutation site of multi-drug resistant *rpoB* gene. Patients should take into account the epidemic characteristics of ETB rifampicin-resistant patients, and effectively develop prevention and control measures. In addition, there are still also some shortcomings in this study. For example, Xpert test was only carried out in Nantong at the end of 2018, resulting in a gap between the mutation data of rifampicin resistance in older adult people and the data of real older adult people, and the analysis conclusion may not be rigorous enough.

## Conclusion

The prevention and treatment effect of ETB patients in Nantong City was significant, and the older adult patients with rifampicin resistance were a problem that needed attention. However, it still has some deficiencies in this study. For example, Nantong has only carried out Xpert testing since the end of 2018, which led to a gap between the mutation data of rifampicin resistance in the older adult and the drug resistance data of the real older adult, and the analysis conclusion may not be rigorous enough.

## Data Availability

The raw data supporting the conclusions of this article will be made available by the authors, without undue reservation.
